# Wireless powering solution for implantable electronics based on ultra-low frequency magnetic energy focusing

**DOI:** 10.1093/nsr/nwae140

**Published:** 2024-04-09

**Authors:** Haoyu Wang, Xinge Yu

**Affiliations:** Department of Biomedical Engineering, City University of Hong Kong, China; Department of Biomedical Engineering, City University of Hong Kong, China

Implantable electronic devices play a crucial role at the frontiers of human health monitoring, disease diagnosis and medical treatment. With the development of their functionality, powering strategies have become a critical issue. However, with existing powering solutions, batteries have a limited lifetime [1]; self-powered devices exhibit unstable and constrained output [2]; ultrasound and near-infrared light-based wireless energy transfer are easily absorbed or reflected in biological tissues and thus quickly attenuated, leading to thermal damage [3,4]; low-frequency magnetic fields decay quickly over distance, and thus the corresponding wireless magnetic energy transfer is restricted by the short effective distance and low power efficiency [5]; and as for magnetic fields with a higher working frequency, such as electromagnetic field radiation or magnetic resonance coupling, the transferred energy attenuates rapidly in bio-tissues, making it difficult to provide power to implantable electronic devices in the deep layer [6].

**Figure 1.**
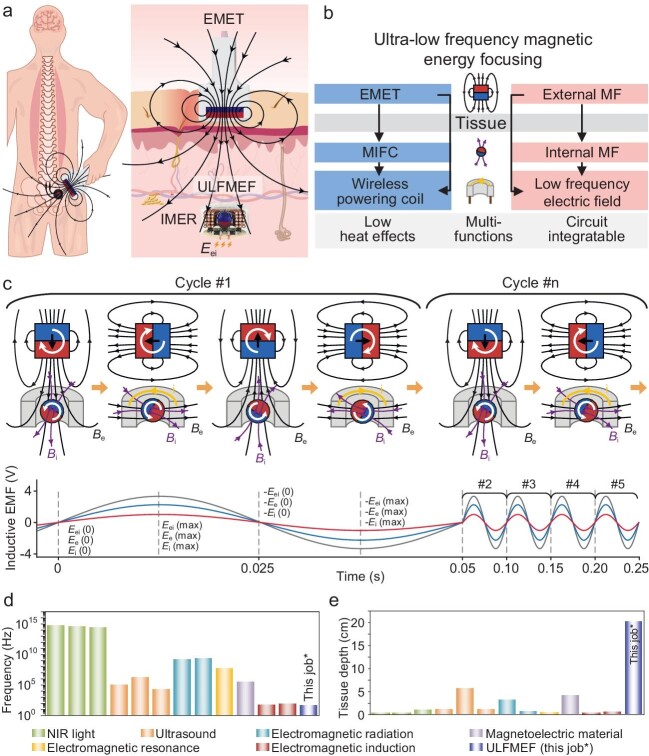
Wireless powering solution enabled by ultra-low frequency magnetic energy focusing. (a) The overall illustration of wireless power transfer in the ULFMEF system through a magnetic field, which can be applied for implantable optical and electrical stimulation. The ULFMEF system consists of an EMET and IMER. (b) Illustration of the scheme and mechanism of the ULFMEF system. (c) The detailed process of synergistic power transmission between the EMET and MIFC. (d and e) Comparison between the ULFMEF and other reported wireless energy transfer technologies, in working frequency and tissue depth. Reprinted with permission from ref. [7].

Recently, published in *National Science Review*, Li *et al.* report a solution to overcome this issue and put forward an ultra-low frequency magnetic energy focusing (ULFMEF) methodology to achieve highly effective wireless energy transfer, which is applicable for deep tissues [7]. As shown in Fig. 1a and b, the researchers design a portable external magnetic energy transmitter (EMET) to generate an external low-frequency magnetic field (<50 Hz) for driving the implantable magnetic energy receiver (IMER). Inside the IMER, there is a magnetism internally focusing core (MIFC) that is driven by the external magnetic field, and which synchronously rotates with it to generate an internal magnetic field. This low-frequency varying internal magnetic field and the initial external magnetic field interact with the coil structure in the IMER, causing it to generate changing magnetic flux and thus induce an electromotive force. This working mechanism is illustrated in Fig. 1c, which addresses the low energy transfer efficiency shortage in low-frequency magnetic energy transfer. The induced electromotive force is directly proportional to the magnetization intensity and frequency, and inversely proportional to the distance. With an external load, the IMER can achieve a significant output of 4–15 mW, with high stability and durability. Compared to other wireless energy transfer technologies, and benefiting from having a low-frequency magnetic field (Fig. 1d), ULFMEF exhibits relatively slow signal attenuation in biological tissues. In particular, ULFMEF shows good wireless energy transfer capability across multiple kinds of materials without high magnetic permeability. As a result, ULFMEF has lower energy loss and thermal damage, making it highly suitable for powering implantable devices in the deep tissues of biological organisms, where its effective range extends up to 20 cm (Fig. 1e).

Furthermore, the IMER demonstrates good biosafety, as mice implanted with the IMER maintained a healthy state in the experiment period, which lasted for several weeks. To demonstrate the effectiveness of ULFMEF, researchers wirelessly powered an implanted optogenetic stimulator in mice, enabling the functionality of optogenetic neuromodulation through optical stimulation. In addition, researchers wirelessly powered an implanted rechargeable-battery-integrated circuit, achieving electrical pulse stimulation. This work has tremendous potential in biomedical applications.

In summary, this novel strategy provides a groundbreaking wireless energy transfer solution based on low-frequency magnetic energy focusing that could provide a solution to the problem of power supply in deep tissues. The considerable output, long effective transmission distance and limited heat generation of this method have the potential to address the current power supply issues of implantable electronic devices. In the near future, ULFMEF systems are expected to find widespread application in the next generation of implantable sensors or therapeutic systems.
